# Fellow-Workers and the Roll of Honour

**Published:** 1915-08-21

**Authors:** 


### ROUND THE WORLD.

[The Editor invites Early Information and Contributions Marked "Fellow-Workers."

### Fellow-Workers and the Roll of Honour.

LIEUTENANT OSWALD ROBERT KELLY, a medical officer of the Red Cross, who went abroad early in the war, and who has lately been given the Cross of the Légion d'Honneur and the Military Cross by the French Government, is a former Westminster Hospital student. Qualifying in 1910, be became an M.B., B.S. Lond., with honours in the following year, and subsequently held the post of house surgeon at Westminster and also at the Royal National Orthopædic Hospital.

CAPTAIN JOHN FITZGERALD GWYNNE, R.A.M.C., recently killed in action on the Western Front, graduated M.B., Ch.B. at Sheffield University in 1911, having been associated all his life with Sheffield, where his father, the late Dr. Charles Nelson Gwynne, had resided for many years, and was after qualification for a time R.M.O. to the Royal Infirmary there. On the outbreak of war the late officer joined the R.A.M.C., and went out with one of the earliest drafts, to distinguish himself on many occasions by gallant conduct in the field, for which he won the D.C.M., was mentioned three times in dispatches, and had, it is understood, been recommended for further distinction when he fell at his post.

MEDICAL men who have been given temporary commissions whilst serving with the new war hospitals include: Drs. W. J. N. Vincent (lieutenant-colonel), D. Gillespie (major), and J. M. Mathieson (major), in connection with the Wharncliffe War Hospital. At the Beaufort War Hospital Drs. R. A. Beaver and T. Mill will serve as majors. It has also been announced that whilst with the County of London War Hospital Dr. P. A. Peall will have temporary rank as major, and Dr. C. F. F. McDowall will rank as captain during his service at the Moss Side Military Hospital.

MR. STANLEY RITSON, B.Sc. Lond., F.R.C.S. Eng., who recently joined the R.A.M.C., and has been appointed specialist in operative surgery to the Grantham Military Hospital, distinguished himself as a student at King's College and Hospital. In addition to winning many scholarships and prizes, he was awarded honours at the B.Sc. Lond., and was appointed demonstrator of physiology and histology at St. Thomas's Hospital, after having held a like post at King's College. His post-graduate appointments include those of house surgeon both at King's College Hospital and at the Evelina Hospital.

DR. JOHN MOORCROFT MCCLOY, M.D., D.P.H., one of the Irish doctors who is going to the Continent with the St. John Ambulance Brigade Base Hospital at Etaples, is assistant tuberculosis officer for Belfast. He was formerly R.M.O. at the City Fever Hospital, Purdysburn.

COLONEL GEORGE LOVELL GULLAND, R.A.M.C., who has just accepted a post as consulting physician to the troops operating in the Mediterranean, with rank as indicated, will, it is understood, be stationed at Malta. In private life he is known to a wide circle as one of the most eminent Scottish physicians of the day, being professor of medicine at Edinburgh University and a member of the staff both at the Royal Infirmary and at the Victoria Consumption Hospital, Edinburgh. Colonel Gulland, who is an M.D., C.M., F.R.C.P., B.Sc. Edin., qualified in 1886.

DR. ISABELLA GALLOWAY EMSLIE, who is taking up work at the Scottish Women's Hospital at Troyes, is an assistant medical officer at Edinburgh Royal Asylum, Morningside. A student of the Edinburgh Medical College for Women, she graduated at the University in 1910, two years later becoming an M.D. Edin., with honours. Her past appointments include those of resident physician to the Edinburgh Royal Hospital for Sick Children and assistant medical officer at Larbert Mental Hospital.

RECENTLY we had to deplore the death at the post of duty of a son of Mr. William Thorburn, F.R.C.S., of Manchester. That eminent surgeon is now himself leaving this country for military work, having been appointed consultant (in surgery) to the Mediterranean Expeditionary Force. Colonel Thorburn, as he will now be, is professor of clinical surgery at Manchester University and senior surgeon to the Manchester Royal Infirmary. He qualified in 1884, taking the M.B. and B.S. degrees of London University and winning three gold medals at his final examinations. Subsequently Mr. Thorburn became an M.D., B.Sc. Lond., and F.R.C.S. Eng.

MR. THOMAS SCALES CARTER, who has been appointed consulting dental surgeon to the 2nd Northern General Hospital at Leeds, qualified as a dental surgeon in 1876. by taking the licensing diploma of the Royal College of Surgeons of England. He was for many years honorary dental surgeon to the Leeds General Infirmary and lecturer on dental surgery at Leeds University.

IT is understood that Dr. Arthur Maxwell Nicholson Pringle, medical officer of health for Ipswich, who has been given a commission as captain in the R.A.M.C., will serve within the borough and continue to carry out his municipal work. Captain Pringle studied medicine at Owens College and became an M.B., C.M. Edin. in 1894.

WORKING with Surgeon-General Lenthal Cheatle, R.N., Dr. Paul Fildes and Dr. L. W. Rajchman have tested with considerable success a new antiseptic mixture, which, it may be fervently hoped, will still further minimise the sufferings of our gallant wounded. Reporting favourably to the *Lancet* (July 24, 1915), these investigators describe their innovation as a mixture of the dye malachite green with corrosive sublimate.

SURGEON-GENERAL CHEATLE, R.N., is so well known to-day that his distinguished career scarcely needs recalling to the readers of a journal of administrative medicine. His services have been at the disposal of the Admiralty since the early days of the war, and latterly he has held a commission carrying high rank. In time of peace Surgeon-General Cheatle was surgeon to King's College Hospital, and was connected with the Royal Navy as examiner for the medical department and member of the Admiralty Departmental Committee on the R.N. medical service.

DR. PAUL FILDES, M.B., B.C. Cantab., is assistant bacteriologist to the London Hospital, and an authoritative writer on his special subject, his investigations in various directions having been of first-class importance. He qualified by graduating in surgery at Cambridge in 1909.

